# 3D-OSEM versus FORE + OSEM: Optimal Reconstruction Algorithm for FDG PET with a Short Acquisition Time

**DOI:** 10.1055/s-0043-1774418

**Published:** 2023-09-13

**Authors:** Keisuke Tsuda, Takayuki Suzuki, Kazuhito Toya, Eisuke Sato, Hirofumi Fujii

**Affiliations:** 1 Department of Radiological Technology, Faculty of Health Science, Juntendo University, Tokyo, Japan; 2 Division of Functional Imaging, Exploratory Oncology Research and Clinical Trial Center (EPOC), National Cancer Center, Japan; 3 Department of Radiology, Tohto Clinic, Tokyo, Japan; 4 Department of Radiology, International University of Health and Welfare Mita Hospital, Tokyo, Japan

**Keywords:** positron emission tomography, short acquisition time, reconstruction, ordered subsets expectation-maximization, Fourier rebinning

## Abstract

**Objective** In this study, we investigated the optimal reconstruction algorithm in fluorodeoxyglucose (FDG) positron emission tomography (PET) with a short acquisition time.

**Materials and Methods** In the phantom study, six spheres filled with FDG solution (sphere size: 6.23–37 mm; radioactivity ratio of spheres to background = 8:1) and placed in a National Electrical Manufacturers Association phantom were evaluated. Image acquisition time was 15 to 180 seconds, and the obtained image data were reconstructed using each of the Fourier rebinning (FORE) + ordered subsets expectation-maximization (OSEM) and 3D-OSEM algorithms. In the clinical study, mid-abdominal images of 19 patients were evaluated using regions of interest placed on areas of low, intermediate, and high radioactivity. All obtained images were investigated visually, and quantitatively using maximum standardized uptake value (SUV) and coefficient of variation (CV).

**Results** In the phantom study, FORE + OSEM images with a short acquisition time had large CVs (poor image quality) but comparatively constant maximum SUVs. 3D-OSEM images showed comparatively constant CVs (good image quality) but significantly low maximum SUVs. The results of visual evaluation were well correlated with those of quantitative evaluation. Small spheres were obscured on 3D-OSEM images with short acquisition time, but image quality was not greatly deteriorated. The clinical and phantom studies yielded similar results.

**Conclusion** FDG PET images with a short acquisition time reconstructed by FORE + OSEM showed poorer image quality than by 3D-OSEM. However, images obtained with a short acquisition time and reconstructed with FORE + OSEM showed clearer FDG uptake and more useful than 3D-OSEM in the light of the detection of lesions.

## Introduction

Positron emission tomography (PET)/computed tomography (CT) test with fluorodeoxyglucose (FDG) is one of the most important diagnostic imaging examinations in clinical oncology. 1 2 3 Although high-performance PET/CT scanners equipped with semiconductor detectors have currently been introduced in clinical practice, PET/CT scanners with scintillators are still popular worldwide.

Conventional PET/CT scanners with scintillators require a long acquisition time and their image quality is easily degraded by body motion.

Our previous study demonstrated that the segmental acquisition method using a Fourier rebinning (FORE) + ordered subsets expectation-maximization (OSEM) reconstruction algorithm could suppress the effects of body motion and improve the image quality of FDG PET, 4 but the effects of this reconstruction algorithm on image data obtained with a short acquisition time have not been well investigated yet. Therefore, we aimed to develop a reconstruction algorithm for a short acquisition time so that it can also be applied to dynamic studies and, by obtaining image data repeatedly with a short scan time, we can obtain PET images from image data that are not affected by body motion, even when body movements occur. A 3D-OSEM algorithm is another reconstruction algorithm for 3D-acquired image data. In 3D-OSEM algorithms, every type of correction of the 3D-acquired image data is performed within the calculation loop of successive approximations, resulting in good quality images. 5 Although previous studies have reported that 3D-OSEM algorithm can provide higher image quality than FORE + OSEM algorithm, 6 7 they did not study image data obtained with a short acquisition time.

In this study, we studied the usefulness of the FORE + OSEM and 3D-OSEM algorithms, and investigated the optimal reconstruction algorithm in FDG PET with a short acquisition time.

## Materials and Methods

### Phantom Study

#### Image Acquisition and Reconstruction

We used a PET/CT scanner with scintillators (Discovery ST Elite Performance; GE Healthcare, Milwaukee, Wisconsin, United States) and an National Electrical Manufacturers Association/International Electrotechnical Commission (NEMA/IEC) body phantom into which six spheres of internal diameters 6.23, 7.86, 10, 13, 22, and 37 mm were placed with their centers all in the same plane. A 70-cm-long scattering phantom was placed adjacent to the NEMA/IEC body phantom. The background area of the NEMA/IEC body phantom was filled with a 3.0 kBq/mL of FDG solution. The concentration of radioactivity in the background area was set to match that of the back muscles of 20 actual patients. 4 The spheres were filled with FDG solution so that the ratio of radioactivity concentration in the spheres to that of the background area was 8:1, in accordance with the MEMA NU-2 2018 standard. 8 The scattering phantom was filled with FDG solution with radioactivity of 66.0 MBq. 8

Image acquisition was started when the centers of the hot spheres in the phantom were aligned with the center of the field of view (FOV) in the body axis. List-mode acquisition was performed in 3D mode, and image data of 12 frames were acquired using a 15 seconds acquisition frame time. Each acquisition was repeated six times. The obtained data were rearranged to produce image data of 15, 30, 60, 120, and 180 seconds. The image with acquisition time of 180 seconds was used as the reference image.

The acquired image data were reconstructed using FORE + OSEM 9 10 and 3D-OSEM (VUE Point Plus, GE Healthcare) algorithms. For the FORE + OSEM algorithm, the numbers of subsets and iterations were set to 15 and 4, respectively. Gaussian filters with full width at half maximum (FWHM) values of 3.91 and 4.29 mm were used as the pre- and postfilters, respectively. For the 3D-OSEM algorithm, the numbers of subsets and iterations were set to 21 and 5, respectively, and a Gaussian filter of FWHM of 4.29 mm was used as the postfilter. These number of iterations and subsets are routinely used in clinical practice. Attenuation correction, 11 scatter correction, 12 and random corrections were performed by methods used routinely in clinical practice.

#### Data Analysis

**Fig. 1:**
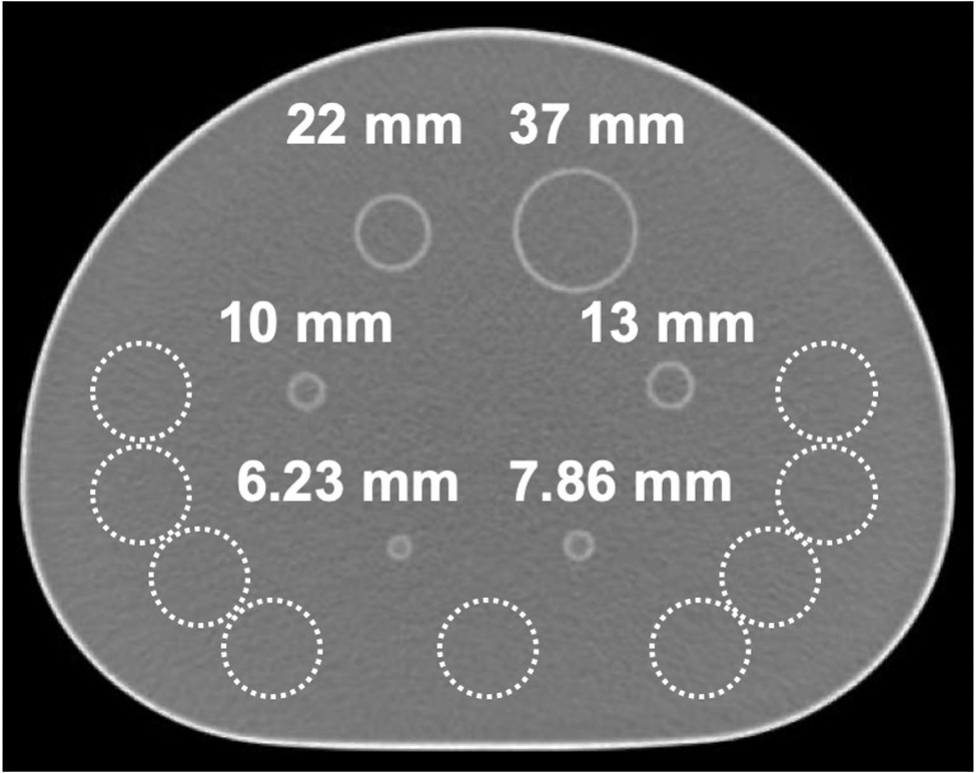
Regions of interest (ROIs) placed on a phantom image. Nine ROIs for calculating the coefficient of variation were placed in the background area ( *dotted lines* ).

The reconstructed PET images were evaluated quantitatively and visually. In the quantitative assessment, one region of interest (ROI) was positioned on each hot sphere and nine ROIs were placed in the background area. ROIs for hot spheres were placed with reference to the CT images. For each PET image, the maximum standardized uptake value (SUV) and the coefficient of variation (CV) 13 were calculated as follows:



where “radioactivity concentration” is the concentration of radioactivity in the hot spheres (units: Bq/g) and “filled dose” is the concentration of radioactivity in the phantom (units: Bq); and



where “S.D.” is the standard deviation of the pixel values in nine 30-mm ROIs placed in the background area around the centers of the hot spheres on each of three slices, and “average” is the average pixel value in the nine ROIs ( Fig. 1 ).

Maximum SUV and CV values of images reconstructed with FORE + OSEM and 3D-OSEM were compared between the reference image (acquisition time: 180 seconds) and those with other acquisition times. The effects of acquisition time on these quantitative indices were statistically analyzed by Dunnett's test. A *p* -value of less than 0.05 was considered significant.

In the visual assessment, three experienced nuclear medicine physicians evaluated the images using the five-point scoring method described by Strobel et al. 14 In the phantom study, the detectability of hot spheres of diameter 7.86, 10, and 22 mm was assessed for all images using the following scale: 1, very difficult to recognize; 2, rather difficult to recognize; 3, undecided; 4, rather easy to recognize; 5, very easy to recognize. In both the FORE + OSEM and 3D-OSEM reconstructed images, the score for the 22 mm-sphere on the reference image was judged to be 5.

The results of the visual assessments were statistically evaluated using Bonferroni test. The significance level was set as *p* = 0.05. Interobserver agreement was assessed using Kendall's W, in R software (R Development Core Team, Vienna, Austria). 15 16

### Clinical Study

#### Patient Data

Patients' PET image data obtained using the same PET/CT scanner. Totally 14,748 PET/CT tests performed by this scanner were saved in the PACS (Picture Archiving and Communication System) server. We reviewed these data of patients whose medical records showed clear evidence of no abdominal lesions and found that additional 3D list-mode acquisition of the mid-abdomen was added in 63 tests to avoid image distortion by body motion. A radiological technologist checked the reconstructed images and selected 19 patient image sets that showed no degradation by body motion. An experienced nuclear medicine physician reviewed these 19 image sets and confirmed that the mid-abdominal images were not affected by body motion. The patients were 12 males and 7 females. In the additional scan, image data of 12 frames with acquisition time of 15 seconds were obtained. Patients fasted for at least 6 hours prior to the examination so their blood-sugar levels would be less than 150 mg/dL at the initiation of scanning. Table 1 lists the characteristics of the patients. We selected image data of the mid-abdominal area including the kidneys with axial FOV of 15.7 cm for 47 slices because images in this range are minimally affected by respiratory motion.

**Table 1:** Patient data summary

Age	62 ± 15 years
Height	163 ± 7 cm
Weight	60 ± 8 kg
Injected activity	317 ± 9 MBq
Time of image acquisition postinjection	93 ± 14 min

#### Data Analysis

The image data acquired in list mode were processed the same way as those in the phantom study, and image data for 15, 30, 60, 120, and 180 seconds were obtained. The acquired image data were reconstructed using FORE + OSEM and 3D-OSEM algorithms, and the images were evaluated both quantitatively and visually, as in the phantom study.

**Fig. 2:**
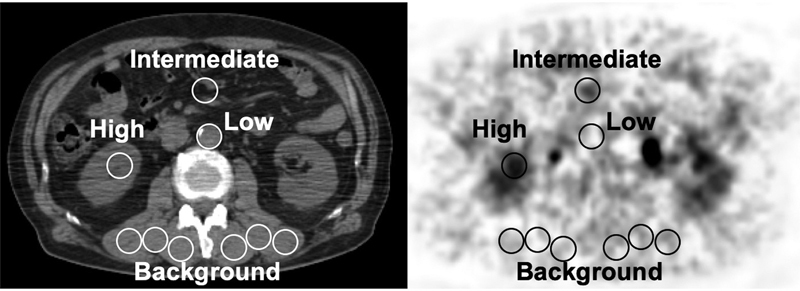
Regions of interest placed on a clinical positron emission tomography/computed tomography image.

In the quantitative assessment, one ROI was placed on an area of low, intermediate, and high radioactivity on the image. The diameter was set to 20 mm for all three ROIs. The ROI for low radioactivity was placed on an area of maximal count 3 to 5 kBq/mL usually on the aorta. The ROI for intermediate radioactivity was placed on an area of maximal count 5 to 7 kBq/mL, usually on the colon. The ROI for high radioactivity was placed on an area of maximal count more than 7 kBq/mL, usually on the renal pelvis. Six background ROIs of diameter 20 mm were placed on the back muscles ( Fig. 2 ).

For each ROI on all images, maximum SUV and CV values were calculated in the same manner as in the phantom study. The results were evaluated statistically by Dunnett's test. A *p* -value of less than 0.05 was considered significant.

In the visual assessment, the same nuclear medicine physicians as in the phantom study reviewed all images using a five-point scoring method 14 : 1, nondiagnostic; 2, poor; 3, undecided; 4, good; 5, excellent. For both the FORE + OSEM and 3D-OSEM reconstructed images, the score of the reference image was judged to be 5.

The detectability of the three types of ROI was assessed, and the score of the high radioactivity area (renal pelvis) of the reference image was considered to be 5 for both the FORE + OSEM and 3D-OSEM reconstructed images. Image quality was also assessed for all images. In both the FORE + OSEM and 3D-OSEM reconstructed images, the score of the reference image with acquisition time of 180 sec was considered to be 5.

The results of the visual assessments were statistically evaluated using Bonferroni test. The significance level was set as *p* = 0.05. Interobserver agreement was assessed using Kendall's W, in R software. 15 16

This retrospective study was approved by the Institutional Review Board (study number 2020-312).

## Results

### Phantom Study

**Fig. 3:**
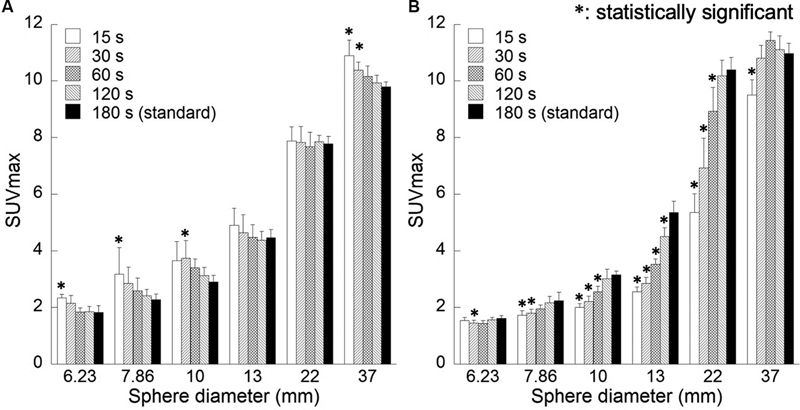
Correlation between sphere diameters and the maximum standardized uptake value (SUVmax) for various acquisition methods. Reconstruction algorithms are as follows: ( **A** ) Fourier rebinning + ordered subsets expectation-maximization (FORE + OSEM) algorithm, ( **B** ) 3D-OSEM algorithm.

Fig. 3 shows the correlation between hot sphere diameter and maximum SUV. Maximum SUV values were generally constant in the FORE + OSEM images, but were higher for short acquisition time and significantly higher for small spheres ( *p* < 0.05). In the 3D-OSEM images, there was a significant decrease in maximum SUV for short acquisition time ( *p* < 0.05).

**Fig. 4:**
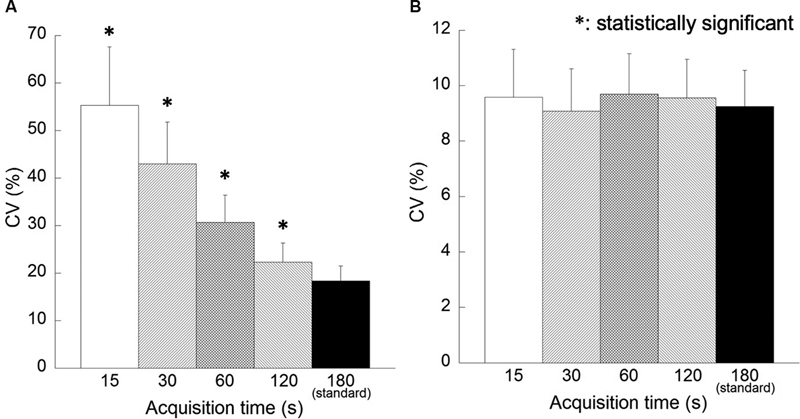
Correlation between acquisition times and the coefficient of variation (CV). Reconstruction algorithms are as follows: ( **A** ) Fourier rebinning + ordered subsets expectation-maximization (FORE + OSEM) algorithm, ( **B** ) 3D-OSEM algorithm.

Fig. 4 shows the correlation between acquisition time and CV. In the FORE + OSEM images, the CV increased when acquisition time was short. Comparison of the reference image and reconstructed images for each acquisition time revealed a significant increase in CV for acquisition time less than 120 seconds. There was no significant difference in CV for any acquisition time in the 3D-OSEM images.

**Fig. 5:**
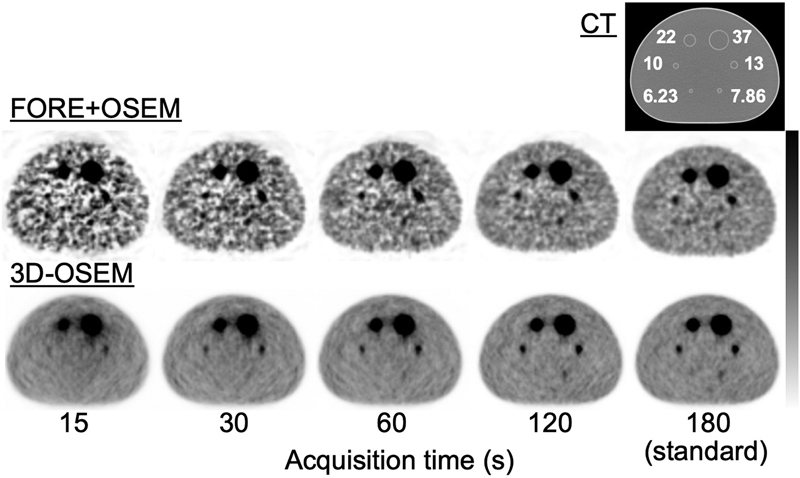
Reconstructed images obtained by various acquisition times. The computed tomographic (CT) image is shown in the *top row* , the reconstructed images obtained by the Fourier rebinning + ordered subsets expectation-maximization (FORE + OSEM) algorithm are shown in the *middle row* , the reconstructed images obtained by the 3D-OSEM algorithm are in the *bottom row* , respectively.

Fig. 5 shows FORE + OSEM and 3D-OSEM images for each acquisition time. The FORE + OSEM images with a short acquisition time showed large variance of background counts and the detectability of small spheres was good. In contrast, 3D-OSEM images with a short acquisition time showed constant variance of background counts but small spheres were not well visualized.

Tables 2 and 3 list the results of the visual assessment of detectability. In the FORE + OSEM images, the detectability of hot spheres of diameter 7.86 and 10 mm was inferior to that of the reference image for acquisition time less than 30 seconds; whereas in the 3D-OSEM images, detectability of these spheres was inferior to that of the reference image for acquisition time less than 60 seconds.

**Table 2:** Visual assessment of phantom images for detectability

Acquisition time (s)	FORE + OSEM		
	7.86 mm	10 mm	22 mm
15	1.3 ± 0.7 a	1.8 ± 1.0 a	4.7 ± 0.5 a
30	1.4 ± 0.8 a	2.8 ± 1.2 a	4.7 ± 0.4
60	1.9 ± 1.1	4.3 ± 0.8	5.0 ± 0.0
120	2.2 ± 1.2	4.6 ± 0.5	5.0 ± 0.0
180 (standard)	2.5 ± 1.3	4.4 ± 0.5	5.0 ± 0.0

Abbreviation: FORE + OSEM, Fourier rebinning + ordered subsets expectation-maximization.

a Statistically significant.

**Table 3:** Visual assessment of phantom images for detectability

Acquisition time (s)	3D-OSEM		
	7.86 mm	10 mm	22 mm
15	1.4 ± 0.8 a	2.3 ± 1.0 a	5.0 ± 0.0
30	1.7 ± 0.9 a	3.3 ± 1.0 a	5.0 ± 0.0
60	2.1 ± 1.0 a	4.1 ± 0.6 a	5.0 ± 0.0
120	2.7 ± 1.1	4.7 ± 0.4	5.0 ± 0.0
180 (standard)	3.1 ± 1.2	5.0 ± 0.0	5.0 ± 0.0

Abbreviation: 3D-OSEM, three-dimensional ordered subsets expectation-maximization.

a Statistically significant.

**Fig. 6:**
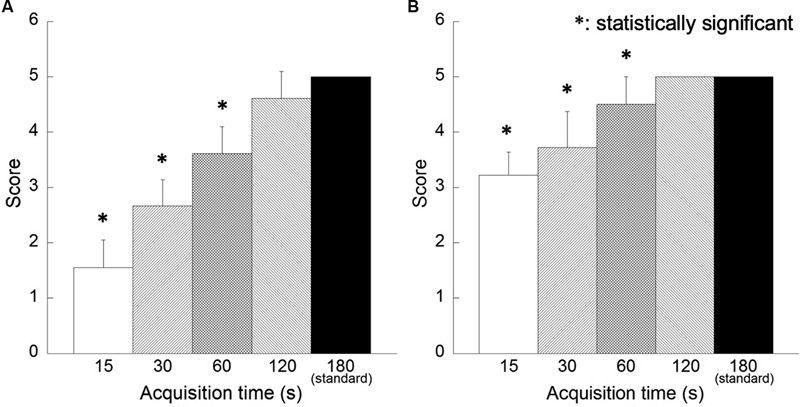
Results of visual assessment of phantom images for image quality. Reconstruction algorithms are as follows: ( **A** ) Fourier rebinning + ordered subsets expectation-maximization (FORE + OSEM) algorithm, ( **B** ) 3D-OSEM algorithm.

Fig. 6 shows the results of the visual assessment of image quality. For each reconstructed image, the image quality for acquisition time less than 60 seconds was judged to be inferior to that of the reference images, and these differences were statistically significant. In terms of overall image quality, however, the 3D-OSEM images scored higher than the FORE + OSEM images. Regarding interobserver agreement among the three nuclear medicine physicians, W-values for detectability and image quality of 0.93 and 0.94, respectively, indicate adequate agreement.

### Clinical Study

**Fig. 7:**
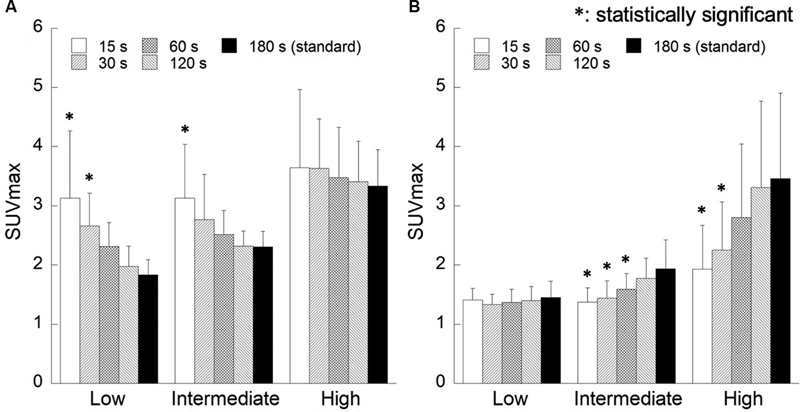
Correlation between the location of the regions of interest and the maximum standardized uptake value (SUVmax) for various acquisition methods. Reconstruction algorithms are as follows: ( **A** ) Fourier rebinning + ordered subsets expectation-maximization (FORE + OSEM) algorithm, ( **B** ) 3D-OSEM algorithm.

Fig. 7 shows the correlation between ROI location and maximum SUV. In the FORE + OSEM images, maximum SUV in the areas of low and intermediate activity was highest for short acquisition time. In the 3D-OSEM images, there was a significant decrease in maximum SUV when acquisition time was short.

**Fig. 8:**
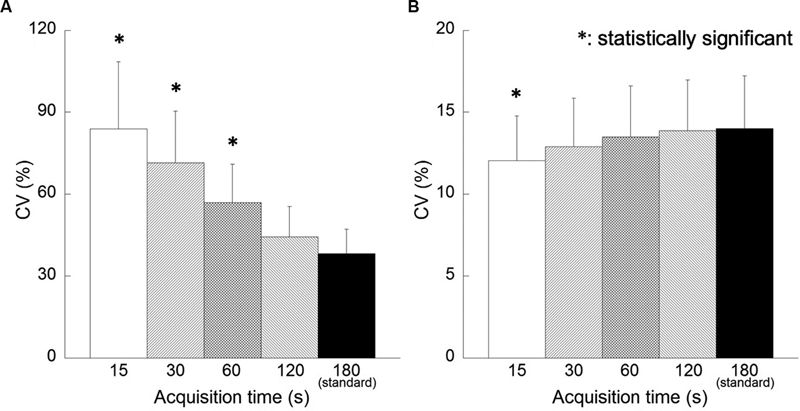
Correlation between the location of the regions of interest and the coefficient of variation (CV). Reconstruction algorithms are as follows: ( **A** ) Fourier rebinning + ordered subsets expectation-maximization (FORE + OSEM) algorithm, ( **B** ) 3D-OSEM algorithm.

Fig. 8 shows the correlation between acquisition time and CV. In the FORE + OSEM images, CV was significantly higher for acquisition time less than 60 seconds in the comparison between the reference image and the reconstructed images for each acquisition time. In the 3D-OSEM images, CV was lower than the reference image when the acquisition time was short. In particular, CV was significantly low in the reconstructed images of acquisition time 15 seconds.

**Fig. 9:**
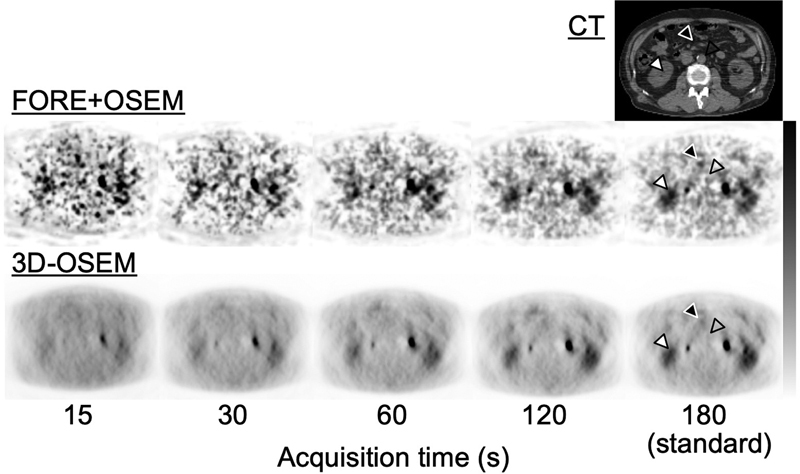
Reconstructed images obtained by various acquisition times. The computed tomographic (CT) image is shown in the *top row* , the reconstructed images obtained by the Fourier rebinning + ordered subsets expectation-maximization (FORE + OSEM) algorithm are shown in the *middle row* , the reconstructed images obtained by the 3D-OSEM algorithm are in the *bottom row* , respectively. The *open* , *black* , and *white arrows* show areas of low, intermediate, and high activity, respectively.

Fig. 9 shows the clinical images of a representative patient. The target sites are indicated by arrows on the CT and PET images. FORE + OSEM images with a short acquisition time showed large variance in background counts, as in the phantom study. Detectability in areas of intermediate and high activity was good even for short acquisition time. On the 3D-OSEM images, variance of background counts was generally constant for each acquisition time; however, the target tissues were not always well visualized when the acquisition time was short.

Tables 4 and 5 list the results of the visual assessment for detectability. In the FORE + OSEM images, detectability was inferior in the intermediate and high activity areas compared with the reference image for acquisition time less than 30 seconds, whereas on the 3D-OSEM images, detectability in these intermediate and high activity areas was inferior compared with the reference image for acquisition time less than 60 seconds.

**Table 4:** Visual assessment of clinical images for detectability

Acquisition time (s)	FORE + OSEM		
	Low	Intermediate	High
15	1.1 ± 0.4*	2.4 ± 1.3 a	2.8 ± 1.4 a
30	1.2 ± 0.6*	2.8 ± 1.1 a	3.6 ± 1.1 a
60	1.9 ± 1.1*	3.5 ± 0.9	4.5 ± 0.8
120	2.4 ± 0.9	3.8 ± 0.7	4.8 ± 0.5
180 (standard)	2.8 ± 0.8	3.9 ± 0.8	5.0 ± 0.0

Abbreviation: FORE + OSEM, Fourier rebinning + ordered subsets expectation-maximization.

a Statistically significant.

**Table 5:** Visual assessment of clinical images for detectability

Acquisition time (s)	3D-OSEM		
	Low	Intermediate	High
15	1.1 ± 0.3 a	1.5 ± 0.7 a	2.5 ± 1.1 a
30	1.2 ± 0.5 _a_	2.0 ± 0.9 a	3.2 ± 1.0 a
60	1.5 ± 0.7 a	2.7 ± 1.0 a	4.0 ± 0.9 a
120	1.8 ± 0.8	3.3 ± 0.9	4.6 ± 0.6
180 (standard)	2.1 ± 0.8	3.7 ± 1.0	5.0 ± 0.0

Abbreviation: 3D-OSEM, three-dimensional ordered subsets expectation-maximization.

a Statistically significant.

**Fig. 10:**
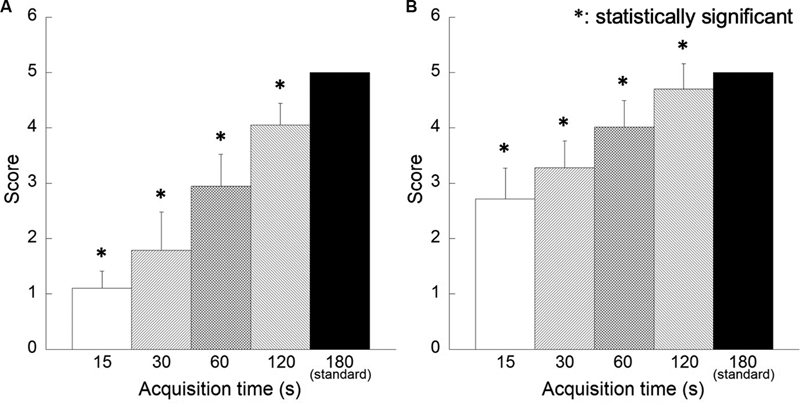
Results of visual assessment of clinical images for image quality. Reconstruction algorithms are as follows: ( **A** ) Fourier rebinning + ordered subsets expectation-maximization (FORE + OSEM) algorithm, ( **B** ) 3D-OSEM algorithm.

Fig. 10 shows the results of the visual assessment for image quality. For each reconstructed image, the quality of images for acquisition time less than 120 seconds was judged to be inferior to that of the reference image; however, in terms of overall image quality, the 3D-OSEM images scored higher than the FORE + OSEM images for all acquisition times. Regarding interobserver agreement among the three nuclear medicine physicians, W-values for detectability and image quality of 0.88 and 0.95, respectively, indicate adequate agreement.

## Discussion

The quality of FDG PET images depends strongly on the obtained counts. Acquisition time must be long enough to obtain images with low statistical noise. 17 In routine clinical practice, we can obtain good quality images in a 3D acquisition when the acquisition time is set to three minutes. 18 In this study, we evaluated the effects of short acquisition time on image quality. In the clinical setting, FDG PET images are usually visually interpreted by nuclear medicine physicians; however, it has been shown that the results of their visual evaluations are not always concordant with quantitative measurements. 19 Therefore, in this study we performed a visual evaluation of the obtained images as well as quantitative measurements.

The image quality of FORE + OSEM images obtained with a short acquisition time was severely degraded by statistical noise. However, maximum SUVs were relatively constant, and the detectability of small spheres was good even for a short acquisition time. In the FORE + OSEM method, the 3D data are first converted to 2D data using the FORE algorithm 9 and all corrections are added to the projection data. These data are then iteratively reconstructed assuming Poisson distribution. 10 Because the raw data are corrected prior to reconstruction, the contrast of the image increases according to the repetition number due to enhancement of the noise component of the projection data. Therefore, even small lesions are likely to be detectable despite the deterioration in image quality due to enhanced image noise.

In contrast, the quality of the 3D-OSEM images did not deteriorate even for short acquisition time, but the maximum SUV decreased significantly, reducing the detectability of small spheres. With the 3D-OSEM algorithm, 3D data are processed directly, without correction. All corrections concerning random coincidence, scatter coincidence, and attenuation corrections are performed during iterative reconstruction, and the reconstruction process is repeated to complete the optimization. Therefore, with the 3D-OSEM algorithm, there is no subtraction process that induces errors as with the FORE + OSEM algorithm. It has been reported that the 3D-OSEM algorithm yields minimal image noise and improves the image quality. 5 In the present evaluation, the image quality of 3D-OSEM images was clearly superior to that of FORE + OSEM images. However, the detectability of small spheres was poor because their small counts were lost among the statistical changes of the background counts when acquisition time was short.

Regarding the quality of images obtained with short acquisition time, 3D-OSEM images scored higher than FORE + OSEM images in our visual assessment. These results correspond with those of the quantitative evaluation. The results were similar even when the spheres were filled with FDG solution so that the ratio of radioactivity concentration in the spheres to that of the background area was set to 4:1 and to 12:1 (data not shown).

The results of quantitative evaluation were similar between the clinical images and those of the phantom study. However, in the clinical FDG PET, CVs of the 3D-OSEM images were significantly lower than those of the FORE + OSEM images for an acquisition time of 15 seconds. In the phantom study, we were able to fill the body phantom with FDG solution uniformly. In the clinical studies, FDG accumulation can change longitudinally according to metabolic activity because activity in the human body is not uniform. In the clinical study, the acquisition started 90 minutes after FDG administration, and the acquisition time was only 3 minutes. As the metabolic activity of glucose in the mid-abdominal areas does not change dynamically under these conditions, the accumulation of FDG was more or less constant, but the visual evaluation of FDG accumulation was difficult in the 3D-OSEM images. This trend was more pronounced in the clinical study than the phantom study.

We obtained unexpected results for the FDG PET with short acquisition time. The FORE + OSEM algorithm yielded noisier images than did the 3D-OSEM algorithm. However, it is important to note that FDG PET examinations are now performed most commonly using a combined PET/CT scanner that provides anatomical information in addition to the counts of FDG accumulation, which enables lesions to be identified easily. Therefore, the enhanced variation in the background count in FORE + OSEM PET images obtained with a short acquisition time would not prevent the detection of small lesions. In this regard, FORE + OSEM is a suitable reconstruction algorithm for use with short acquisition time.

## Conclusion

In this study, we compared FDG PET images reconstructed with each of the FORE + OSEM and 3D-OSEM algorithms after short time acquisition. Three-dimensional OSEM images showed better quality but poorer detectability for small uptake; whereas the quality of FORE + OSEM images was worse, but better detectability for small uptake. As PET/CT combined scanners are now widely available and the superimposed CT images provide the anatomical information to identify locations of small lesions, we consider that the FORE + OSEM algorithm is more suitable for the reconstruction of FDG PET image data acquired with a short time.
